# Radioactivity and radionuclides in deciduous teeth formed before the Fukushima-Daiichi Nuclear Power Plant accident

**DOI:** 10.1038/s41598-021-89910-0

**Published:** 2021-05-14

**Authors:** Atsushi Takahashi, Mirei Chiba, Akira Tanahara, Jun Aida, Yoshinaka Shimizu, Toshihiko Suzuki, Shinobu Murakami, Kazuma Koarai, Takumi Ono, Toshitaka Oka, Joji Ikeyama, Osamu Kaneko, Makoto Unno, Kimiharu Hirose, Takashi Ohno, Yasushi Kino, Tsutomu Sekine, Ken Osaka, Keiichi Sasaki, Hisashi Shinoda

**Affiliations:** 1 Tohoku University Hospital, Tohoku University, 1-1 Seiryo-machi, Aoba-ku, Sendai, Miyagi 980-8574 Japan; 2 Graduate School of Dentistry, Tohoku University, 4-1 Seiryo-machi, Aoba-ku, Sendai, Miyagi 980-8575 Japan; 3 Faculty of Science, University of the Ryukyus, Senbaru, Nishihara, Nakagami, Okinawa 903-0129 Japan; 4 Graduate School of Medical and Dental Sciences, Tokyo Medical and Dental University, 1-5-45 Yushima, Bunkyo-ku, Tokyo, 113-8549 Japan; 5 Collaborative Laboratories for Advanced Decommissioning Science, Japan Atomic Energy Agency, 10-2 Fukasaku, Miharu, Fukushima 963-7700 Japan; 6 Department of Chemistry, Tohoku University, 6-3 Aramaki-aoba, Aoba-ku, Sendai, Miyagi 980-8578 Japan; 7 Sector of Nuclear Science Research, Japan Atomic Energy Agency, 2-4 Shirakata, Tokai, Naka, Ibaraki 319-1195 Japan; 8 The Fukushima Prefecture Dental Association, 6-6 Chugen-cho, Fukushima, Fukushima 960-8105 Japan; 9 Faculty of Dentistry, Ohu University, 31-1 Misumido, Tomitamachi, Koriyama, Fukushima 963-8611 Japan; 10 Institute for Excellence in Higher Education, Tohoku University, 41 Kawauchi, Aoba-ku, Sendai, Miyagi 980-8576 Japan

**Keywords:** Environmental sciences, Environmental social sciences, Risk factors

## Abstract

The Fukushima-Daiichi Nuclear Power Plant (FNPP) accident in March of 2011 released substantial amounts of radionuclides into the environment. We collected 4,957 deciduous teeth formed in children before the Fukushima accident to obtain precise control data for teeth formed after the accident. Radioactivity was measured using imaging plates (IP) and epidemiologically assessed using multivariate regression analysis. Additionally, we measured ^90^Sr, ^137^Cs, and natural radionuclides which might be present in teeth. Epidemiological studies of IP showed that the amount of radioactivity in teeth from Fukushima prefecture was similar to that from reference prefectures. We found that artificial radionuclides of ^90^Sr and ^137^Cs, which were believed to have originated from past nuclear disasters, and natural radionuclides including ^40^ K and daughter nuclides in the ^238^U and ^232^Th series contributed to the generation of radioactivity in teeth. We also found no evidence to suggest that radionuclides originating from the FNPP accident significantly contaminated pre-existing teeth. This is the first large-scale investigation of radioactivity and radionuclides in teeth. The present findings will be indispensable for future studies of teeth formed after the FNPP accident, which will fall out over the next several years and might be more contaminated with radionuclides.

## Introduction

The Fukushima-Daiichi Nuclear Power Plant (FNPP) accident during March 2011 released substantial amounts of radionuclides into the atmosphere, resulting in extensive environmental contamination^1–10^. Radionuclides such as ^90^Sr, are incorporated into developing teeth and remain until the tooth falls out or is extracted^11^. Since the radionuclide content in teeth is thought to parallel the amount of systemic incorporation^12^; we postulated that the amount of internal exposure to radiation in children from Fukushima could be estimated by measuring the radionuclide content in teeth that developed after the FNPP accident.

Precise control data about teeth that developed before the FNPP accident are indispensable to accurately evaluate the nuclear contamination of teeth caused by the FNPP accident. However, few studies have systematically investigated the types and concentrations of radionuclides originally contained in teeth and whether they differ regionally. Thus, accurate control data are needed from before the FNPP accident to serve as the basis for evaluating teeth that developed thereafter.

Several issues needed to be resolved before teeth formed before and after the FNPP accident could be compared. One was the yield of fission products such as ^90^Sr and ^137^Cs in teeth. The presence of these radionuclides in teeth has been reported in connection with past nuclear disasters^13–19^. Small amounts of ^90^Sr and ^137^Cs originated from past nuclear disasters have been found in soil samples from various locations in Japan, including Fukushima prefecture, before the accident^20^. Besides, trace amounts of ^90^Sr and ^137^Cs fallout are still detectable, even though atmospheric nuclear weapons testing was conducted between the 1950s and the 1970s^21^. Low levels of ^90^Sr have been found not only in the natural environment but also in animal teeth that formed before the FNPP accident^22^. However, no systemic data are available on the concentration of such radionuclides in human teeth developed before the accident. Furthermore, whether radionuclide contents in teeth differ between individuals from Fukushima prefecture and those living in other areas is important to determine. Another issue is the types of the radioactivity of natural radionuclides in teeth. Teeth contain natural radionuclides such as ^40^K and daughter nuclides in the ^238^U and ^232^Th series found in the environment^23^. However, the type and concentration of these radionuclides in teeth remain obscure.

Furthermore, even completely formed teeth might have been secondarily contaminated with radionuclides released into the environment after the accident^24^. Radionuclides could be deposited on tooth surfaces and/or incorporated into teeth through pulp via the bloodstream. Secondary contamination could thus be a factor that increased the amount of radioactivity in teeth that formed before the accident. Therefore, whether artificial radionuclides released into the environment after the accident were incorporated into fully formed teeth needed to be determined.

**Figure 1:**
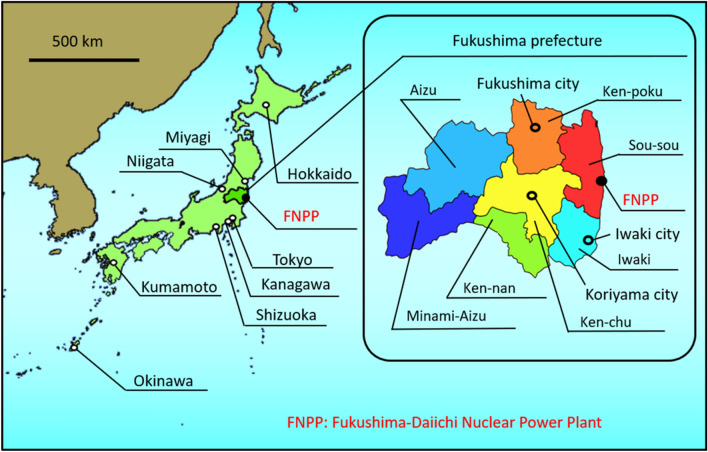
Locations of prefectures and seven administrative districts in Fukushima where deciduous teeth were collected. The maps were modified from open-access base maps freely available for public and academic use (source: https://maps.gsi.go.jp/, from the Geographic Information Authority of Japan).

We collected 4,957 deciduous teeth from children living in the Fukushima area and other areas where the effects of the FNPP accident were considered negligible between 2014 and 2016 (Fig. 1). All teeth were considered to have been formed before the FNPP accident because the calcification of the crowns of deciduous teeth starts at the gestational age of 4 months, is completed by the age of 12 months^25^ and the teeth fall out between the ages of 6 and 12 years. Thus, to collect enough deciduous teeth that formed after the accident will take several more years.

We used imaging plates (IP) to radioactivity expressed in terms of quantum levels (QLs) in deciduous teeth collected from children living in Fukushima and reference prefectures. We then epidemiologically analysed factors that contributed to the detected QL. We measured concentrations of radionuclides that emit gamma rays (^40^ K, ^137^Cs, ^134^Cs, and natural radionuclides in the uranium and thorium series) and beta rays (^90^Sr) using a germanium (Ge) detector and a low-background gas-flow counter, respectively. The present findings should serve as an important foundation by providing control data for future studies on teeth developed after the FNPP accident.

## Results

### Radioactivity in deciduous teeth determined using IP

Figure S1 in supplementary information (SI) shows the radioactivity in all teeth examined. The QLs exceeded the mean + 3 standard deviations (SD) in 24 teeth collected from Fukushima and the other prefectures (19 [0.46%] of 4,130 and 5 [0.60%] of 827 teeth, respectively). Most of the teeth with high QLs were filled with composite resin. Further investigation revealed that some types of composite resin emitted significant beta and gamma rays that resulted in IP with high QLs (data not shown). Since the current study’s objective was to determine whether the amount of radioactivity in natural teeth increased after the FNPP accident, we eliminated all teeth with fillings and/or decayed portions on the target surface. Therefore, 3,814 deciduous teeth with healthy target labial/buccal surfaces were further investigated.

### Measurement of radionuclides in teeth

#### Natural radionuclides in deciduous teeth

Table 1 summarises the results of the gamma ray measurements. The ^214^Pb in ^238^U and ^212^Pb in ^228^Th series, as well as ^40^K, were found within a measurement period of 1 million seconds (11.6 days). The activity concentrations were consistently higher for ^40^K than the other natural radionuclides. Radioactive caesium was not detected during this study’s measurement period.

**Table 1:** Radioactivity of natural radionuclides (mBq/g) in deciduous teeth.

	^214^Pb (U series)	^212^Pb (Th series)	^40^K
Hokkaido (8)	1.8 ± 0.6	0.82 ± 0.24	6.1 ± 3.5
Kumamoto (6)	2.6 ± 0.9	1.50 ± 0.25	10.3 ± 5.6
Shizuoka (6)	2.3 ± 1.1	LTD	7.5 ± 2.8
Kanagawa (5)	3.3 ± 0.9	LTD	7.7 ± 3.7

Data are shown as mean ± counting error. Parentheses show numbers of teeth in a sample. Nuclides were measured in teeth for 1 million seconds (11.6 days). When the counts did not exceed the mean + 3SD of the background, we considered the value as LTD (lower than detection limit). SD, standard deviation.

#### Determination of ^90^Sr in deciduous teeth

Table 2 shows the ^90^Sr findings in deciduous teeth collected from the major cities of Fukushima, Iwaki, and Koriyama in Fukushima prefecture and the reference prefectures, Hokkaido, Niigata, Shizuoka, and Kumamoto (Fig. 1). The amount of ^90^Sr radioactivity detected in teeth ranged from undetectable to 2.05 mBq/g Ca. However, ^90^Sr activity concentrations in teeth did not significantly differ between Fukushima and the reference prefectures.

**Table 2:** Radioactivity of ^90^Sr (mBq/g Ca) in deciduous teeth.

		^90^Sr
Fukushima prefecture	Iwaki city-1 (6)	1.45 ± 0.16
	Iwaki city-2 (8)	1.28 ± 0.15
	Iwaki city-3 (16)	2.05 ± 0.67
	Fukushima city-1 (14)	1.61 ± 0.12
	Fukushima city-2 (16)	LTD
	Koriyama city-1 (13)	1.57 ± 0.12
	Koriyama city-2 (16)	1.77 ± 0.70
Reference prefecture	Hokkaido-1 (9)	1.63 ± 0.13
	Hokkaido-2 (14)	LTD
	Niigata-1 (6)	2.01 ± 0.09
	Niigata-2 (14)	LTD
	Shizuoka-1 (6)	1.95 ± 0.11
	Shizuoka-2 (17)	LTD
	Kumamoto-1 (6)	1.45 ± 0.08
	Kumamoto-2 (16)	1.88 ± 0.73

Data are shown as mean ± counting error. Parentheses show numbers of teeth in a sample. When the counts did not exceed the mean + 3SD of the background, we considered the value as LTD (lower than detection limit). SD, standard deviation.

#### Determination of ^137^Cs in deciduous teeth

**Figure 2:**
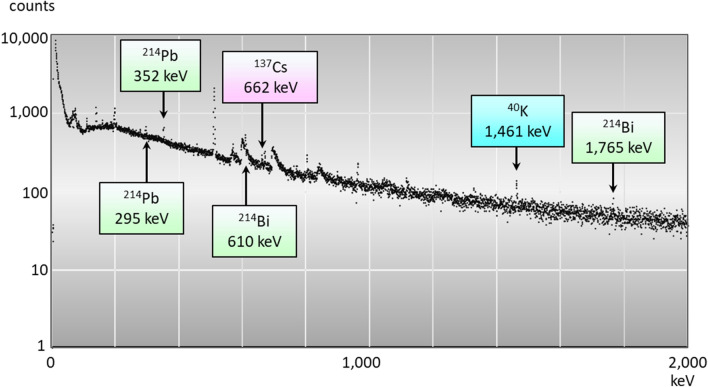
Typical gamma-ray spectrum including the peak of ^137^Cs in deciduous teeth. The ordinate shows counts measured for 3 million seconds (34.7 d).

Since no radioactive caesium was detected during the 1 million seconds (11.6 days) measurement, we increased the duration of measurement (Table 1) to 1.6–3.0 million seconds (18.5–34.7 days). By doing so, we could increase the peak counts (662 keV) and detect trace amounts of radioactive caesium. Figure 2 shows a representative gamma ray spectrum of deciduous teeth measured for 3 million seconds (34.7 days). The ^137^Cs peak should occur at 662 keV. Extending the measurement period to 34.7 days reduced the signal-to-noise ratio and revealed a ^137^Cs peak.

Table 3 shows the amounts of ^137^Cs and other natural radionuclides in teeth from the Fukushima and reference prefectures. The teeth from the Fukushima prefecture were divided into the following 4 groups according to the time they fell out: Fukushima A, before the FNPP accident; Fukushima B: 0–2 after the FNPP accident; Fukushima C, 2–4 after the FNPP accident; and Fukushima D: 4–6 years after the FNPP accident. Trace amounts of ^137^Cs were detected in teeth from Fukushima and the reference prefectures. We detected ^137^Cs in teeth that fell out before and after the FNPP accident in teeth in all groups except group C from Fukushima prefecture, while ^134^Cs was undetectable in all samples.

**Table 3:** Radioactivity of ^137^Cs and natural radionuclides in deciduous teeth (mBq/g).

	^137^Cs	^214^Pb (U series)	^212^Pb (Th series)	^40^K
Fukushima A(12)	0.21 ± 0.07	0.91 ± 0.31	0.39 ± 0.15	7.9 ± 1.5
Fukushima B(23)	0.39 ± 0.03	0.61 ± 0.16	LTD	14.0 ± 0.9
Fukushima C(8)	LTD	LTD	LTD	12.6 ± 3.8
Fukushima D(8)	0.79 ± 0.09	LTD	0.39 ± 0.15	10.9 ± 2.8
Hokkaido (21)	0.14 ± 0.04	0.57 ± 0.14	0.27 ± 0.07	10.2 ± 1.1
Shizuoka (24)	0.07 ± 0.02	0.65 ± 0.16	0.24 ± 0.03	5.4 ± 1.0
Niigata (8)	0.60 ± 0.16	0.65 ± 0.38	LTD	7.9 ± 2.2
Kumamoto (14)	0.25 ± 0.06	0.41 ± 0.18	LTD	8.5 ± 1.0

Data are shown as mean ± counting error. Parentheses show numbers of teeth in a sample. When the counts did not exceed the mean + 3SD of the background, we considered the value as LTD (lower than detection limit). SD, standard deviation. Since radioactive caesium was not detected during the 1 million seconds measurement (11.6 days), we increased the duration of measurement (Table 1) to 1.6–3.0 million seconds (18.5–34.7 days) to increase peak counts (662 keV). The teeth from the Fukushima prefecture were divided into the following 4 groups according to the time they fell out: Fukushima A, before the FNPP accident; Fukushima B, 0–2 after the FNPP accident; Fukushima C, 2–4 after the FNPP accident; and Fukushima D, 4–6 years after the FNPP accident. ^134^Cs was undetectable in all samples.

The natural radionuclides, ^214^Pb in the ^238^U series, ^212^Pb in the ^232^Th series, and ^40^K were detected in teeth, reflecting the results obtained after measuring gamma rays for 11.6 days. The activity concentration was consistently higher for ^40^K than the other natural radionuclides.

### Epidemiology of the radioactivity in deciduous teeth

Table S1 shows associations between various factors and the QLs of teeth determined by multivariate multilevel regression analysis adjusted for several confounding factors.

#### Regional differences

**Figure 3:**
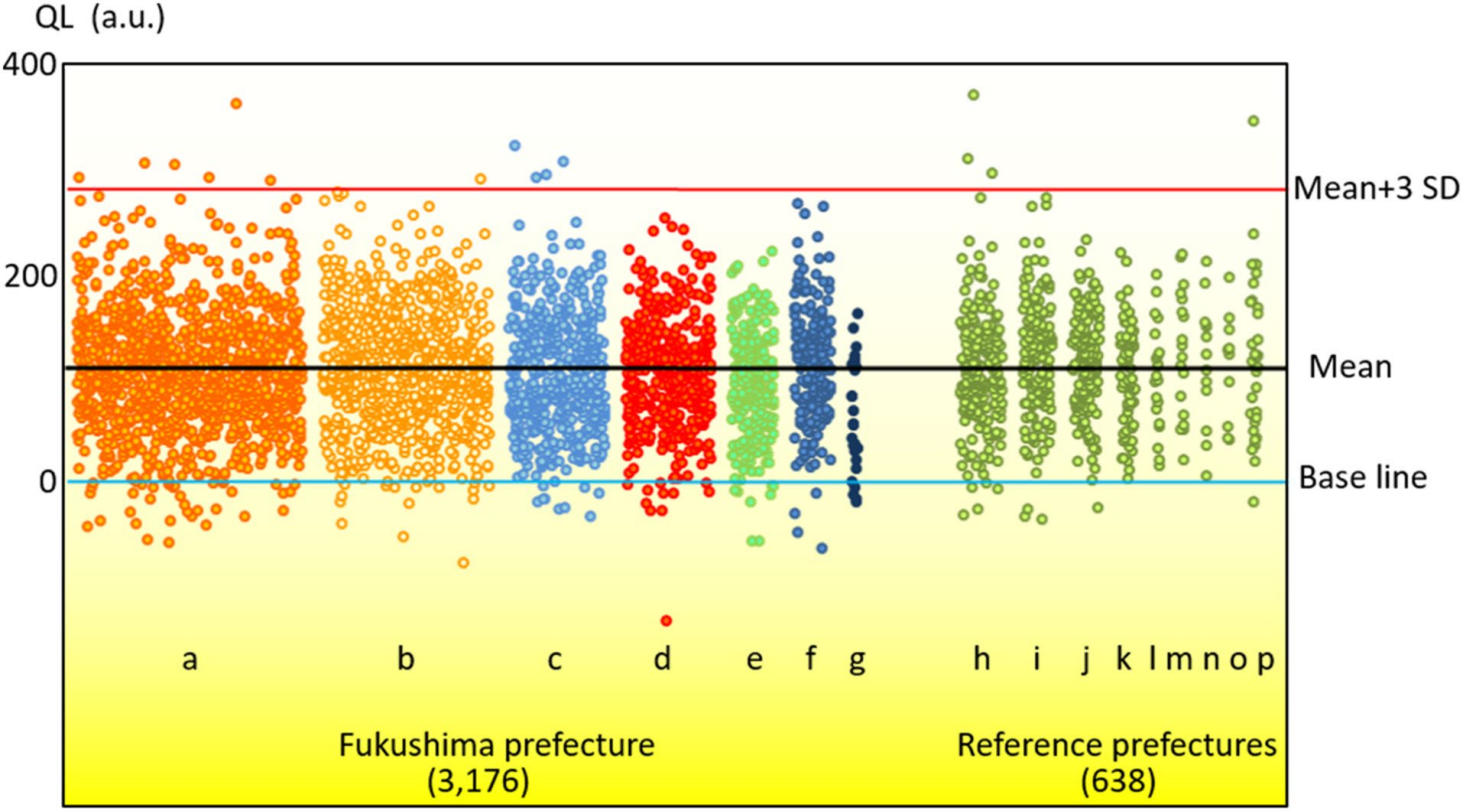
Quantum levels (QL) of deciduous teeth collected from seven districts in Fukushima and those from reference prefectures. The black line shows the mean QL, the red line shows the mean QL + 3 standard deviations (SDs), and the blue line shows the base line. The numbers of teeth are shown in parentheses. (**a**) Ken-poku, (**b**) Ken-chu, (**c**) Iwaki, (**d**) Sou-sou, (**e**) Ken-nan, (**f**) Aizu, (**g**) Minami-Aizu, (**h**) Niigata, (**i**) Shizuoka, (**j**) Hokkaido, (**k**) Kumamoto, (**l**) Miyagi, (**m**) Tokyo, (**n**) Okinawa, (**o**) Kanagawa, (**p**) others. a.u.: arbitrary units.

Figure 3 shows that the QLs of teeth did not significantly differ between the seven districts in Fukushima and the reference prefectures. However, the QL was lower in teeth collected from the Minami-Aizu, compared with the Ken-poku district, which served as the standard. Table S2 shows the mean QLs, SDs, numbers of teeth, and average air dose rates for each district.

#### Types of teeth

**Figure 4:**
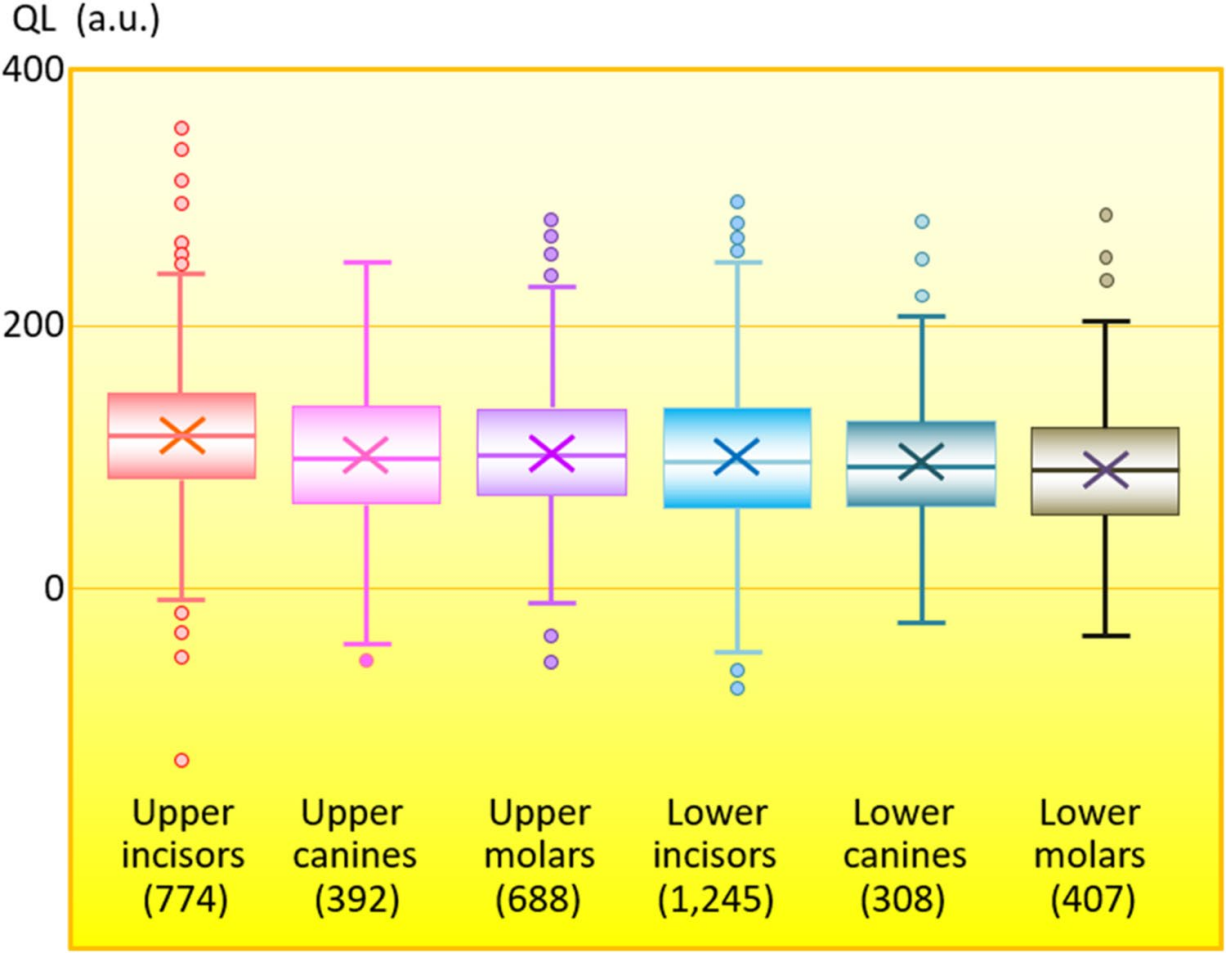
Quantum levels (QL) of various types of teeth. a.u.: arbitrary units. The QL is the highest in the upper incisors (*p* < 0.01). Parentheses show numbers of teeth.

Figure 4 shows that the QL was the highest in a maxillary deciduous incisor, and the mean QL was significantly higher for maxillary than deciduous incisors than for any other types of maxillary or mandibular deciduous teeth (*p* < 0.01).

#### Duration that teeth remained in the oral cavity after the accident

**Figure 5:**
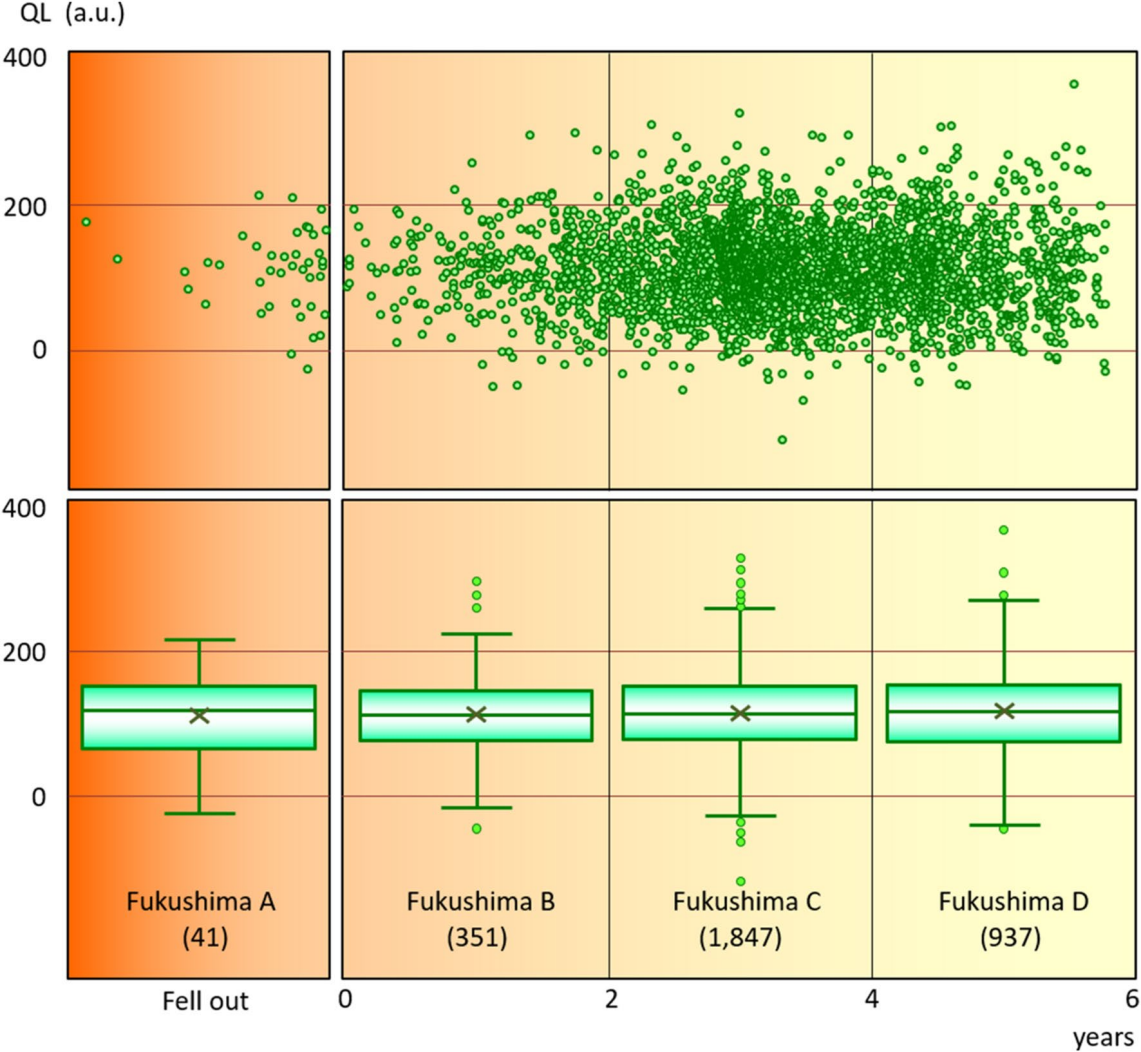
Quantum levels (QL) of teeth in different duration of time teeth remained in the oral cavity after the FNPP accident. Parentheses show numbers of teeth. a.u.: arbitrary units.

We aimed to determine the prevalence of secondary contamination, such as radionuclide deposition on the tooth surface and/or incorporation into the pulp, caused by radionuclides in the environment after the FNPP accident. However, the amount of radioactivity did not significantly correlate with the length of time that teeth remained in the oral cavity after the accident (Fig. 5).

#### Effects of gender, age at the time of the accident, air dose rate, and FNPP accident-related evacuation

None of the above factors were significantly associated with the QLs of the teeth.

## Discussion

We measured radioactivity in deciduous teeth collected between January 2014 and December 2016 using IP, which have become valuable radiation research tools^26–28^. One advantage is that IP have high sensitivity with an excellent linear response ranging over three orders of magnitude. We obtained a good linear correlation (R^2^ = 0.9998), ranging from 0 to 1,000 mBq ^40^K/g (Figure S2). Corresponding to a background of 3 SD, the estimated detection limit of QL was 91.9 (equivalent to 13.1 mBq ^40^K/g in the reference scale. Furthermore, > 100 teeth could be concurrently measured using one IP, thus enabling the assessment of numerous teeth without destruction.

With respect to natural radionuclides, ^226^Ra, a daughter nuclide in the ^238^U series, has been identified in permanent teeth from Japan different concentrations among regions^18^. The activity concentration of ^226^Ra is higher in teeth from inhabitants living in areas of high, compared with low natural background radiation areas in Ramsar^29^. The low-background, well-type Ge detector identified ^214^Pb, a daughter radionuclide in the ^238^U series, in deciduous teeth. We also found trace amounts of ^212^Pb in the ^232^Th series. These findings indicated that the deciduous teeth contained natural radionuclides in both the ^238^U and ^232^Th series. We also identified the ^40^K gamma spectrum, ranging from 6.1 to 10.3 mBq/g, in deciduous teeth. The K contents in tooth enamel and dentin are 0.037%–0.30%^11,30^ and 0.02%–0.04%^11^, respectively, and 0.0117% of K is radioactive (30.4 Bq ^40^K/g K). Here, the values ranging from 6.1–10.3 mBq ^40^K/g, (0.2–0.34 mg K/g (or 0.020–0.034%). Therefore, these K concentrations, extrapolated based on the present results, are similar to those of the previous studies.

Several studies have shown a relationship between radionuclides in teeth and the environment. For example, ^90^Sr was incorporated into human teeth after liquid radioactive waste was released into the Techa River during the early 1950s, resulting in radioactive contamination of the entire Techa River region^13–16^. Additionally, ^90^Sr levels are higher in the deciduous teeth of Swiss children born between 1952 and 2002^17^ and in people who resided in south-west Poland in the 1970s, in association with increased environmental ^90^Sr levels due to nuclear weapon tests, and in teeth of residents after the Chernobyl accident in 1986^17,24,31^. These findings suggest that radioactivity in teeth reflects the environmental amount of radioactivity. Here, we detected small amounts of ^90^Sr that did not significantly differ in deciduous teeth from Fukushima and reference prefectures (^90^Sr activity concentrations: < 2.05 and < 2.01 mBq/g Ca, respectively). To the best of our knowledge, ^90^Sr concentrations in Japanese human deciduous teeth have not been reported. However, reports have described ^90^Sr concentrations in tissues from livestock before the FNPP accident. The activity concentration of ^90^Sr in cattle bones from Hokkaido decreased from ~ 70 mBq/g Ca in 1966, to 26 mBq/g Ca in 2008^32^. Koarai et al.^22^ detected a ^90^Sr concentration of 14.7 ± 7 mBq/g Ca in the teeth of cattle sacrificed in 2013 in Iwate prefecture, which is 250 km north of the FNPP and believed to be unaffected by the accident. The authors concluded that ^90^Sr might have originated from past nuclear weapon tests conducted between 1950 and 1970. Moreover, ^90^Sr has been detected in the soil of Fukushima prefecture, as well as other locations in Japan before the FNPP accident^1,20^. Thus, we concluded that the ^90^Sr detected herein did not originate from the FNPP accident but rather from environmental contamination caused by prior atmospheric nuclear weapon tests.

Like ^90^Sr, ^137^Cs is a common component of local and global radioactive fallout and was released following various nuclear disasters. Increased levels of ^137^Cs were reported in the deciduous teeth of children from south-west Poland in 1970s^19^. However, our knowledge about the amount of ^137^Cs in teeth is still limited. We detected a small amount of ^137^Cs in teeth collected from Fukushima and reference prefectures after precise measurements lasting from 18.5 to 34.7 days using a low-background Ge detector. The levels of ^137^Cs were one order of magnitude lower than those of ^90^Sr. That ^137^Cs did not originate from the FNPP accident is likely because it was detected in teeth that had fallen out before the accident and in teeth from areas where the effects of the accident were negligible. Furthermore, all examined teeth were formed before the FNPP accident, and ^134^Cs was undetectable. The FNPP accident released equal amounts of ^134^Cs and ^137^Cs into the environment, and they might still be detectable. Some reports have suggested that the Japanese environment contained ^137^Cs before the FNPP accident^33–36^. For example, the increased ^137^Cs concentration in foods after atmospheric nuclear tests continues to decrease^33–35^. Therefore, the findings of ^137^Cs and ^90^Sr in teeth formed before the accident need to be considered in future studies of teeth developed after the accident.

The QLs of teeth from Fukushima and the reference prefectures did not significantly differ even though the air dose rate considerably differed among districts after the FNPP accident. However, the QL was lower in teeth from the Minami-Aizu, than other districts. The reason for this is unknown. However, geological information obtained from a map of Japan’s natural radioactivity before the accident shows relatively low natural radioactivity in the area where Minami-Aizu is located^37,38^. This might explain the lower QLs of teeth from Minami-Aizu.

The QLs of upper incisors was slightly, but significantly higher than that of the other types of teeth, regardless of whether teeth were from maxillary or mandibular bones. Beta rays are the main contributors to the generation of the QL. The self-absorption of beta rays occurs to a relatively large degree due to the high density of teeth. Therefore, IP mainly detect beta rays that are emitted by structures proximal to the tooth surface. Hence, differences in QLs are related to differences in radiation levels at the tooth surface. The surfaces of the upper incisors are more easily exposed to the atmosphere than that of other types of teeth. Therefore, natural radionuclides, such as ^222^Rn in the ^238^U series, in the atmosphere could be deposited on the surface of upper incisors more easily.

Radionuclides are incorporated into teeth either during tooth formation or via secondary contamination after teeth are completely formed. Secondary contamination results when radionuclides from food, water, or the atmosphere, are deposited on the tooth surface or incorporated via pulp. In fact, permanent teeth formed before the Chernobyl accident contain a large amount of radioactive strontium^24^. All teeth examined in the present study were also formed before the FNPP accident. Therefore, we presumed that the increase in the QLs was caused by secondary contamination in the present study. The QLs of teeth did not increase according to the length of time that teeth remained in the oral cavity or air dose rate. These findings suggested that teeth were not significantly affected by secondary contamination by radionuclides released into the environment after the FNPP accident. Therefore, future studies of teeth formed after the FNPP accident should consider that any increase in QLs is related to radionuclides originating from the accident.

Studies that systemically focus on radioactivity and related radionuclides in human teeth are scarce. The present findings revealed that the amount of radioactivity in human deciduous teeth exceeded the background level, even after IP were shielded with lead to minimise natural background radiation. We also detected natural radionuclides, including ^214^Pb in the ^238^U series, ^212^Pb in the ^232^Th series, and ^40^K, as well as artificial radionuclides, including ^90^Sr and ^137^Cs. Moreover, ^40^K and ^90^Sr (including its daughter nuclide ^90^Y) apparently played important roles in the IP response because their concentrations and beta ray energy were relatively higher than those of the other radionuclides. Therefore, when investigating radioactivity levels in teeth formed after the FNPP accident, the knowledge that teeth originally contained radioactive nuclides is important to consider.

The present study systematically measured amounts of radioactivity in thousands of teeth collected from Fukushima prefecture and reference prefectures. Our results indicated that teeth formed before the FNPP accident originally contained natural and artificial radionuclides unrelated to the FNPP accident. Furthermore, regional amounts and types of radioactivity in teeth collected from Fukushima and reference prefectures did not significantly differ. We found no evidence to suggest that radionuclides originating from the FNPP accident significantly contaminated pre-existing teeth. Our findings may serve as important control data for future studies on teeth formed after the FNPP accident, which will proceed over the next several years. To the best of our knowledge, this is the first large-scale epidemiological investigation of radioactivity in teeth, and the data will be important for evaluating changes in radioactive substances in teeth associated with the FNPP accident and environmental changes.

## Materials and methods

### Collection of teeth

We collected 4,957 deciduous teeth that had fallen out or were extracted during dental procedures from children residing in Fukushima and other prefectures between January 2014 and December 2016 (Fig. 1). The Tohoku University Graduate School of Dentistry, Fukushima Prefecture Dental Association, and School of Dentistry, Ohu University signed agreements regarding the study’s purpose, analysis of the collected teeth, and the means of returning results to the donors on November 5, 2013. The study was performed in accordance with the Declaration of Helsinki and informed consent was obtained from the legal guardians of all participants. The study protocol was approved by the Ethics Committees of Tohoku University Graduate School of Dentistry, the Fukushima Prefecture Dental Association, and the School of Dentistry, Ohu University on January 31, 2013 (Approval No. 23–19); December 17, 2013; and March 18, 2014 (Approval No. 97); respectively. Teeth were collected by dentists in clinics located mainly in Fukushima prefecture. Dentists at Ohu University Dental Hospital, and those in other prefectures invited to participate by Fukushima Prefecture Dental Association, also assisted with tooth collection. The teeth were cleaned with deionised water and sent to the Center for Environmental Dentistry, Tohoku University Graduate School of Dentistry, along with background information about each donor. Medial, distal, labial, lingual, and occlusal surfaces of the teeth were photographed using a stereomicroscope, Leica EZ4D, Wetzlar, Germany) and securely stored.

### Radioactivity measurements of deciduous teeth

#### Measurements using IP

We measured radioactivity in deciduous teeth using BAS-MS 2040 IP (FUJIFILM Corp., Tokyo, Japan). The teeth were positioned such that their labial/buccal surfaces faced the IP surface, then IP cassettes were placed in a shielding box made of lead with iron plates for 4 weeks to block natural background radiation (Figure S2). Details of how the teeth were positioned on the IP are described in the SI. The amount of radioactivity in each tooth was determined as QLs using a Fuji FLA-7000 bio-imaging analyser (FUJIFILM Corp.) and ImageQuant TL 8.1 (GE Healthcare). We used nine IPs to measure radioactivity in thousands of samples. However, the sensitivity of the IP varied, and the QLs differed even when the same sample was measured using different IP. Therefore, we normalised the QLs obtained from different IPs by placing a reference scale of potassium chloride on each one to calibrate the radioactivity values (Figure S2). The methods used to normalise the QLs are detailed in the SI.

#### Measurement of natural radionuclides and radioactive caesium

We measured gamma rays emitted from radionuclides in teeth using a low-background, well-type, CANBERRA GCW3023 Ge detector (Mirion Technologies, San Raymon, CA, USA) with a shield of 20-cm thick lead blocks^39^. Since the amount of radioactivity of individual teeth was extremely low; we measured samples of 5–8 teeth packed to fill 7–8 mm of plastic vials with a diameter of 14 mm using forceps to minimise voids. The radioactivity of ^214^Pb in ^238^U series, ^212^Pb in ^232^Th series, ^40^K, ^137^Cs, and ^134^Cs in teeth was determined as peaks corresponding to the following: 295 and 352 keV for ^214^Pb; 239 for ^212^Pb; 1,461 for ^40^K; 605 for ^134^Cs; and 662 for ^137^Cs. The measurement period was approximately 1 million seconds (11.6 days), and the estimated overall measurement errors were < 15% for the determination of ^40^K.

For radioactive caesium measurements in the teeth from groups Fukushima A–D (Table 3 and Fig. 5), radionuclides were measured in teeth for 1.6–3.0 million seconds (18.5 to 34.7 days) to increase peak counts (662 keV) and detect trace amounts of radioactive caesium. Radioactive caesium in tooth samples (n = 8–23) selected from groups Fukushima A–D was determined in descending order of QLs in each group. We measured samples from 8–23 teeth packed to 35 mm high. Teeth from reference prefectures were similarly measured.

The heights of samples in the vial ranged from 7–35 mm with a density of 0.8–1.0 g/cm^3^. The height of tooth sample greatly contributes to the counting efficiency during gamma ray measurement. CaCO_3_ mixed with mineral powder (CRM 42–2 standard reference material, New Brunswick Laboratory, Argonne, IL, United States)^40^ was used to account for the variation in sample height. The relationship between counting efficiency and height of standard material at different energy gamma ray is shown in Figure S3. The difference in counting efficiency in regions above 200 keV, less than 35 mm in height, and a diameter of 14 mm were < 10%.

#### Determination of ^90^Sr in deciduous teeth

Deciduous teeth do not generally contain measurable amounts of ^90^Sr. Therefore, ^90^Sr was determined in samples of 5–10 teeth (> 2 g per sample) as described by Koarai et al^22^. Briefly, teeth were incinerated at 450 °C, followed by chemical separation of Sr from Ca using the fuming nitric acid method. Then, Ra and Pb were chemically removed via co-precipitation with BaCrO_4_, while Y was removed via co-precipitation with Fe(OH)_3_. Thereafter, the growth of ^90^Y from ^90^Sr was monitored by measuring beta rays emitted from ^90^Sr and its daughter ^90^Y using a low-background LBC-4201B gas-flow counter (Hitachi-Aloka Medical, Ltd.). Then, activity concentration of ^90^Sr was calculated. The certificated reference material (fish bone, JSAC 0471, the Japan Society for Analytical Chemistry) was used as a quality control sample for ^90^Sr measurement.

### Epidemiology of radioactivity in deciduous teeth

#### Regional differences at the time of the FNPP accident

We investigated regional differences between the QLs of teeth from Fukushima and reference prefectures and seven administrative districts in Fukushima prefecture (Fig. 1).

#### Types of deciduous teeth examined

We determined whether the QL differs among incisors, canines, and molars from maxillary and mandibular bones.

#### Differences related to duration teeth in the oral cavity after FNPP accident

We examined teeth that were formed before the FNPP accident. Therefore, radionuclides that originated from the FNPP accident were probably not incorporated into these teeth during their formation. However, secondary contamination with radionuclides can occur even after teeth are completely formed. Therefore, we assessed whether the QLs increased depending on how long the teeth remained in the oral cavity after the accident. We referred to the report by The Japanese Society of Pedodontics^41^ for the timeline of deciduous tooth eruption and deciduation.

#### Gender differences

We investigated the influence of gender on the QLs of teeth.

#### Differences in age at the time of the FNPP accident

We examined whether age at the time of the accident affected the QLs of teeth.

#### Air dose rate

Secondary contamination could be caused by environmental radionuclides. Therefore, we assessed correlations between the QLs of teeth and air dose rates in the areas^42^ where the donor children resided. Air dose rates used in each area are described in the SI.

#### Evacuation due to FNPP accident

We investigated the effects of evacuation on the QLs of the teeth.

### Statistical analysis

We performed a multilevel regression analysis to determine whether the seven factors mentioned above could affect the QLs of deciduous teeth. Of the 3,814 teeth with a healthy surface on the IPs, we eliminated 7 teeth for which enough information about the seven aforementioned factors was not available. Teeth were nested in individuals in multilevel models (3,807 teeth from 2,746 children). Confounding by multiple factors was considered using a multivariate model. Values with *p* < 0.05 were considered significantly different. Data were statistically analysed using Stata/MP version 15 (Stata Corp., College Station, TX, USA).

## Supplementary Information

Supplementary Information.

## Data Availability

The datasets generated during and/or analysed during the current study are available from the corresponding author on reasonable request.
