# Supplementary material for: Radioactivity and radionuclides in deciduous teeth formed before the Fukushima-Daiichi Nuclear Power Plant accident
Source: Sci Rep. 2021 May 14;11:10335. doi: 10.1038/s41598-021-89910-0 (PMC8121844; doi:10.1038/s41598-021-89910-0)
Supplement: Supplementary file 1 — Supplementary Information. [file 41598_2021_89910_MOESM1_ESM.pdf]

Supplementary Information

Radioactivity and radionuclides in deciduous teeth formed before the Fukushima-Daiichi Nuclear Power Plant accident

Atsushi Takahashi[1], Mirei Chiba[2], Akira Tanahara[3], Jun Aida[4], Yoshinaka Shimizu[2], Toshihiko Suzuki[2], Shinobu Murakami[2], Kazuma Koarai[5], Takumi Ono[6], Toshitaka Oka[7], Joji Ikeyama[8], Osamu Kaneko[8], Makoto Unno[8], Kimiharu Hirose[9], Takashi Ohno[9], Yasushi Kino[6], Tsutomu Sekine[10], Ken Osaka[2], Keiichi Sasaki[2] & Hisashi Shinoda*[2]

[1]Tohoku University Hospital, Tohoku University, 1-1 Seiryo-machi, Aoba-ku, Sendai, Miyagi 980-8574, Japan

[2]Graduate School of Dentistry, Tohoku University, 4-1 Seiryo-machi, Aoba-ku, Sendai, Miyagi 980-8575, Japan

[3]Faculty of Science, University of the Ryukyus, Senbaru, Nishihara, Nakagami, Okinawa 903-0129, Japan

[4]Graduate School of Medical and Dental Sciences, Tokyo Medical and Dental University, 1-5-45 Yushima, Bunkyo-ku, Tokyo 113-8549, Japan

[5]Collaborative Laboratories for Advanced Decommissioning Science, Japan Atomic Energy Agency, 10-2 Fukasaku, Miharu, Fukushima 963-7700, Japan

[6]Department of Chemistry, Tohoku University, 6-3 Aramaki-aoba, Aoba-ku, Sendai, Miyagi 980-8578, Japan

[7]Sector of Nuclear Science Research, Japan Atomic Energy Agency, 2-4 Shirakata, Tokai, Naka, Ibaraki 319-1195, Japan

[8]The Fukushima Prefecture Dental Association, 6-6 Chugen-cho, Fukushima, Fukushima 960-8105, Japan

[9]Faculty of Dentistry, Ohu University, 31-1 Misumido, Tomitamachi, Koriyama, Fukushima 963-8611, Japan

[10]Institute for Excellence in Higher Education, Tohoku University, 41 Kawauchi, Aoba-ku, Sendai, Miyagi 980-8576, Japan

Contents

Setting teeth on imaging plates **S3**

Normalization of sample quantum levels **S3**

Air dose rates applied to each area **S4**

Figure S1 **S5**

Figure S2 **S6**

Figure S3 **S7**

Table S1 **S8**

Table S2 **S9**

Setting teeth on imaging plates

A 7.5-μm-thick Kapton polyimide film (The Nilaco Co., Ltd., Tokyo, Japan) was placed between the teeth and the imaging plate (IP) to avoid contaminating the IP. The IP surfaces were covered with Saran wrap plastic film (Asahi Kasei Home Products Corp., Tokyo, Japan) to provide added protection from contamination and moisture. The back of each sample and spaces between samples were covered and filled, with JM SILICONE dental silicon impression paste (J. Morita Corp., Tokyo, Japan) and Cemedine 8060 silicon sealant (Cemedine Co., Ltd., Tokyo, Japan), respectively, to form plates approximately 10 mm thick. The IP were then placed in exposure cassettes (GE Healthcare Japan, Tokyo, Japan).

Normalization of sample quantum levels

Naturally occurring potassium contains 0.0117% radioactive ^{40}K. Different activity concentrations of ^{40}K powder, ranging from 0 to 1,000 mBq/g, were obtained by evaporating solutions containing different ratios of KCl and NaCl. Thereafter, plastic tubes (diameter, 7 mm; wall thickness, 3 mm) were packed with each standardized powder, at a density of 1.24 g/cm^3, then closed by covering each end with polyimide film. These reference scales enabled the normalization of the measured quantum levels (QL) of each sample to the equivalent value of ^{40}K (mBq/g, at a thickness of 3 mm).

We used the following regression formula:

$$(1)\ \mathrm{Y} = \mathrm{aX} + \mathrm{b},$$

which was obtained from the relationship between activity concentrations (units, Bq; known values) and the QL of ^{40}K in each reference scale (Figure S1). However, the QL

cannot be expressed as Bq of a radioisotope such as ^{40}K, because teeth might contain various radioisotopes that emit different types of radiation. Furthermore, the ratios of radioisotope concentration in the samples were unknown. Therefore, we converted the Bq for the sample to QL using the averaged equation:

$$(2)\ Y = a_m X + b_m,$$

of nine equations ($Y = a_1X + b_1$, $Y = a_2X + b_2$, $Y = a_3X + b_3$…, and $Y = a_9X + b_9$). Since we used nine IP, the values of a_m and b_m were calculated as:

$$(3)\ a_m = (a_1 + a_2 + a_3 + a_4 + a_5 + a_6 + a_7 + a_8 + a_9)/9,$$

and

$$(4)\ b_m = (b_1 + b_2 + b_3 + b_4 + b_5 + b_6 + b_7 + b_8 + b_9)/9.$$

We regarded the standardized QL as representative. We also measured the background radiation QL in every IP measurement. Thereafter, we subtracted the background values from the measured QL of the teeth, and further analysed the results. The standard deviation of the background QL was 30.6, which corresponded to 4.36 mBq/g of ^{40}K in the reference scale.

Air dose rates used for each area

The air dose rate used for each area was calculated as the mean of the air dose rates measured at all primary schools within each local administrative education district (5–20 schools) in the Fukushima prefecture. Radioactivity was measured in primary schools in the evacuation zone on April 18 or 19, 2011[1] and between April 5 and 7, 2011 in schools in the Fukushima prefecture located outside of the evacuation zone[42]. We also used air dose rates measured on April 1, 2011 at radiation monitoring posts nearest the respective offices of the reference prefectures[20].

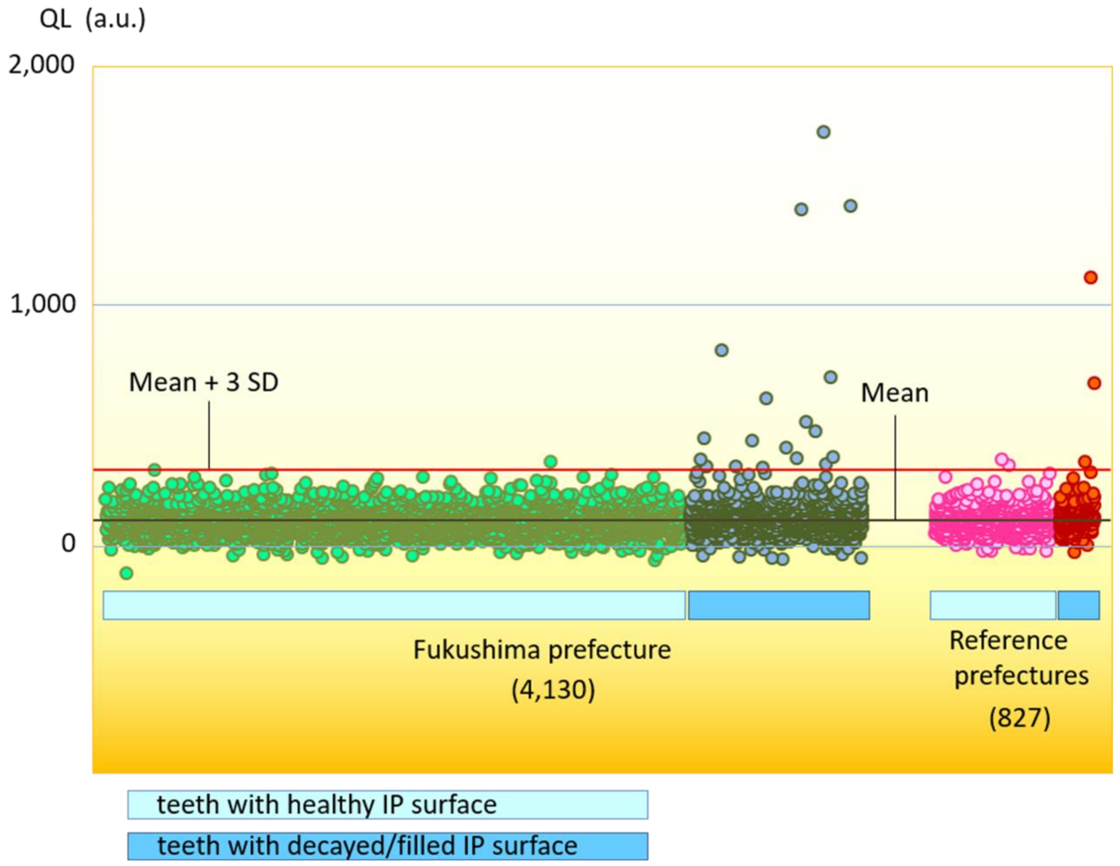

Figure S1. Distribution of the quantum levels (QL) of all teeth collected. The black line indicates the mean QL, and the red line indicates the mean QL + 3 standard deviations (SD). The bars at the bottom show whether the measurements were from healthy (light blue) or decayed/filled (dark blue) teeth. Numbers of teeth are shown in parentheses. a.u., arbitrary units; IP, imaging plate.

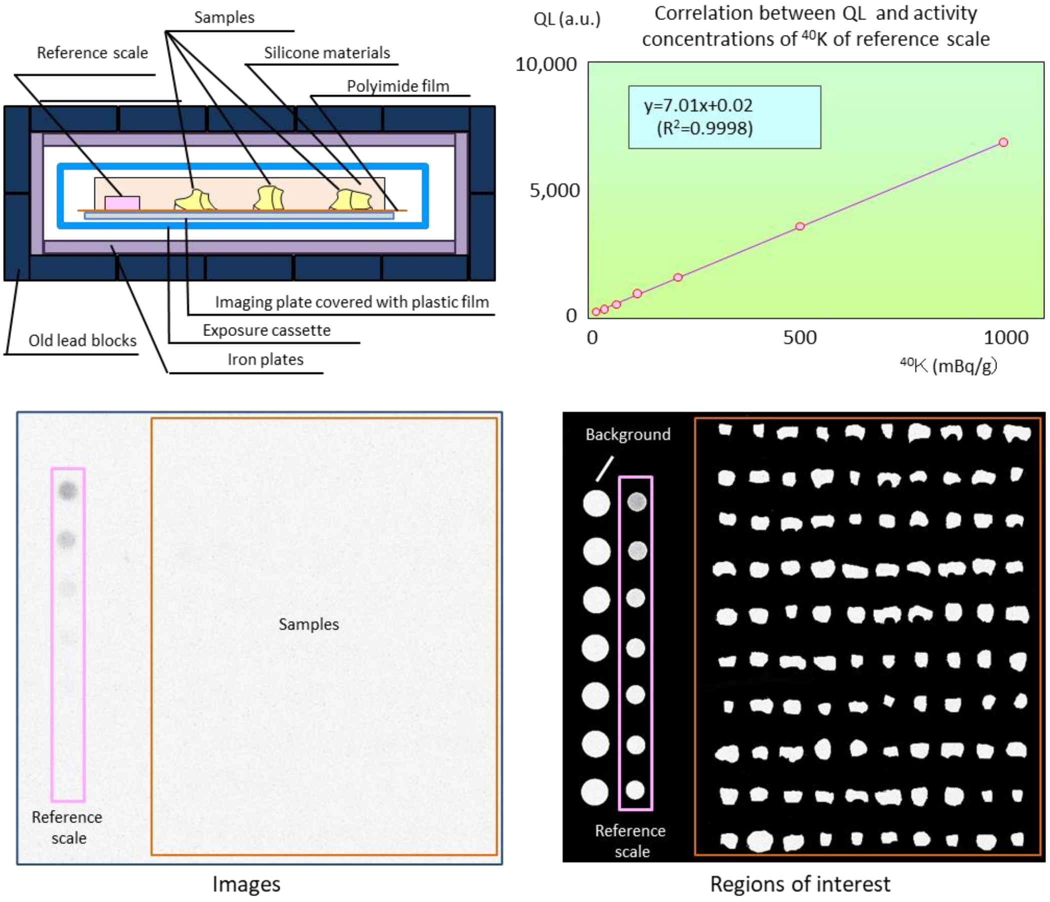

Figure S2. Method for exposure of radioactivity using an imaging plate (IP). Top left: schematic representation of teeth on the IP. Top right: the correlation between quantum levels (QLs) and the activity of different ^{40}K concentrations in the reference scale. Bottom left: images of teeth obtained using the IP and the reference scale. Bottom right: regions of interest of the teeth on the IP. a.u., arbitrary units.

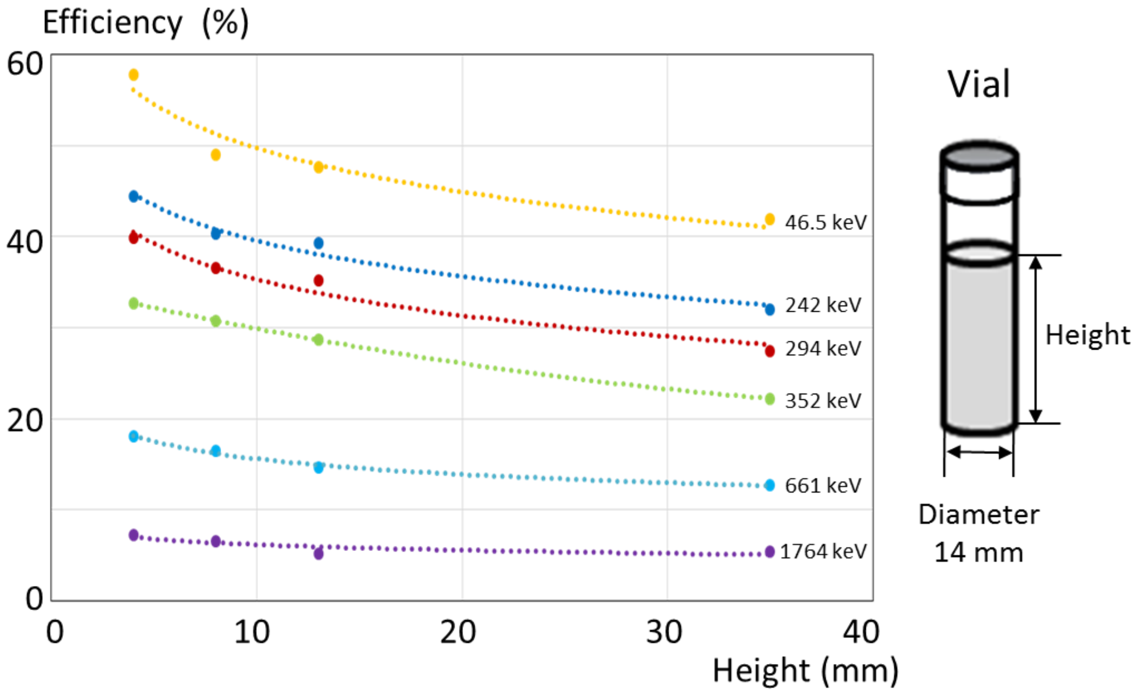

Figure S3. Relationship between the height of standard reference material and counting efficiency.

Table S1. Association of factors with QL of teeth determined using multivariate multilevel regression (n = 3,807 teeth from 2,746 children).

	B (95 % confidence interval)	P
Region (Ref: Ken-poku)		
Ken-chu	9.42 (-0.26; 19.11)	0.057
Iwaki	11.36 (-4.18; 26.89)	0.152
Sou-sou	4.03 (-6.10; 14.17)	0.435
Ken-nan	-1.60 (-13.80; 10.59)	0.797
Aizu	11.70 (-5.69; 29.09)	0.187
Minami-Aizu	-28.83 (-54.97; -2.70)	0.031
Niigata	60.42 (-58.80; 179.63)	0.321
Shizuoka	67.79 (-51.58; 187.15)	0.266
Hokkaido	64.32 (-54.97; 183.61)	0.291
Kumamoto	52.48 (-67.07; 172.04)	0.390
Miyagi	40.13 (-17.75; 98.00)	0.174
Tokyo	76.82 (-50.39; 204.03)	0.237
Okinawa	60.58 (-60.99; 182.14)	0.329
Kanagawa	51.64 (-73.51; 176.79)	0.419
Others	82.95 (-34.88; 200.79)	0.168
Gender (Ref: Male)		
Female	1.85 (-2.86; 6.57)	0.442
Unknown	2.52 (-2.59; 7.63)	0.333
Age (y) at time of FNPP accident (Ref: 0–2 y)		
3–4	-2.72 (-9.50; 4.05)	0.430
5–6	-3.82 (-13.38; 5.74)	0.433
7–17	-5.02 (-17.56; 7.53)	0.433
Days that teeth remained after FNPP accident (quartile, Ref: 0–947 days)		
948–1172	2.01 (-3.99; 8.02)	0.511
1173–1488	-7.15 (-14.20; -0.11)	0.046
1489–2103	-1.95 (-10.56; 6.66)	0.657
Radiation air dose rate, (Ref: 0–0.099)		
0.100–0.499	14.75 (-90.19; 119.70)	0.783
0.500–0.999	10.32 (-95.76; 116.41)	0.849
1.000–1.999	17.07 (-88.61; 122.76)	0.752
2.000–2.999	24.35 (-81.82; 130.52)	0.653
3.000–9.999	19.91 (-86.46; 126.29)	0.714
10.00–99.99	25.99 (-81.08; 133.06)	0.634
Teeth type (Ref: Maxillary deciduous incisors)		
Maxillary deciduous canines	-12.62 (-20.91; -4.33)	0.003
Maxillary deciduous molars	-11.04 (-18.58; -3.51)	0.004
Mandibular deciduous incisors	-17.33 (-23.06; -11.61)	0.000
Mandibular deciduous canines	-17.22 (-25.21; -9.24)	0.000
Lower jaw deciduous molars	-24.24 (-32.12; -16.37)	0.000
Residential relocation (Ref: none)		
Within Fukushima prefecture	0.44 (-5.37; 6.24)	0.882
Moved into Fukushima prefecture	-48.65 (-107.24; 9.94)	0.104
Moved outside Fukushima prefecture	-31.76 (-86.11; 22.59)	0.252

Table S2. Quantum levels (QLs) of deciduous teeth collected from seven districts in the Fukushima and reference prefectures.

	QL (a.u)	Air dose rate*
Fukushima prefecture (3,176)		
Ken-poku (1,088)	105.7 ± 57.2	2.52
Ken-chu (805)	107.8 ± 55.2	1.71
Iwaki (465)	103.8 ± 55.5	0.86
Sou-sou (411)	102.8 ± 51.7	4.34
Ken-nan (204)	94.6 ± 55.3	1.13
Aizu (175)	109.7 ± 56.3	0.35
Minami-Aizu (28)	65.8 ± 52.6	0.13
Reference prefecture (638)		
Niigata (198)	106.2 ± 56.1	0.05
Shizuoka (142)	116.2 ± 56.1	0.04
Hokkaido (131)	113.2 ± 46.6	0.03
Kumamoto (68)	99.9 ± 47.7	0.03
Miyagi (22)	98.0 ± 49.2	0.10
Tokyo (18)	122.0 ± 59.1	0.10
Okinawa (13)	113.7 ± 58.6	0.02
Kanagawa (10)	109.7 ± 51.0	0.07
others (36)	113.9 ± 72.5	-

The data represent the means ± standard deviations. The parentheses indicate the numbers of teeth. a.u., arbitrary units. *Average air dose rate in April 2011 (μSv/h).
